# CD200-CD200R Interaction: An Important Regulator After Stroke

**DOI:** 10.3389/fnins.2019.00840

**Published:** 2019-08-07

**Authors:** Xu Zhao, Jing Li, Haitao Sun

**Affiliations:** ^1^Department of Neurosurgery, The National Key Clinical Specialty, The Engineering Technology Research Center of Education Ministry of China, Guangdong Provincial Key Laboratory on Brain Function Repair and Regeneration, Zhujiang Hospital, Southern Medical University, Guangzhou, China; ^2^The Second Clinical Medical College, Southern Medical University, Guangzhou, China; ^3^Key Laboratory of Mental Health of the Ministry of Education, Guangdong-Hong Kong-Macao Greater Bay Area Center for Brian Science and Brain-Inspired Intelligence, Southern Medical University, Guangzhou, China

**Keywords:** stroke, microglia, neuroinflammation, CD200-CD200R interaction, clinical potential

## Abstract

The high mortality and morbidity rate of stroke is a chronic problem that plagues human society. The activation of microglia is one of the principal reasons why neuroinflammation induces cerebral dysfunction. Because of their vital functions in the regulation of neuroinflammation, microglia constitute an important target for stroke. Given that there is an innate self-preservation mechanism between neurons and microglia, the transmembrane glycoproteins on the surface of their membranes, namely CD200 and CD200R, have become a popular topic of research. Numerous studies have demonstrated that CD200-CD200R interaction, microglial activation, and poststroke neuroinflammatory damage are inextricably linked. In this review, we describe the above relationship from a new perspective. We specifically focus on neuroinflammation after stroke. The role of crosstalk of CD200-CD200R inhibitory immune ligand receptors in immune regulation will also be illustrated. Thus, we will see how poststroke injury can be influenced by the CD200-CD200R crosstalk. Finally, we will discuss the possibility of clinical application of the result of CD200-CD200R interaction to manage neuroinflammatory injury after stroke.

## Introduction

Stroke with high mortality and morbidity rates have a high incidence worldwide and threatens human health and quality of life. It is a devastating disease and continues to play an important role in current research in the field of medicine. Portegies, from the Epidemiology Department of Erasmus MC University Medical Center, found that between 1990 and 2012, 74.3% of patients with their first-ever stroke died (919/1237), but only 53.8% of stroke-free participants died (2654/4928) (Portegies et al., 2016). Stroke is classified into two types: ischemic stroke and hemorrhagic stroke. The former accounts for over 80% of stroke cases (Weinstein et al., 2010). In recent years, several therapeutic strategies for stroke have been used in clinical practice. Unfortunately, most patients are unable to obtain timely and proper treatment because of the narrow therapeutic time window, and only 3.4–5.2% of patients receive timely treatment during the acute phase (Writing Group et al., 2016). During the subacute and chronic phases, it is therefore quite essential to discover other combinations of treatments to facilitate the functional rehabilitation of patients with stroke (Kanazawa et al., 2017).

Following the use of the magnetic resonance imaging (MRI) techniques at the cellular and molecular level, researchers have obtained a new understanding of the inflammatory reaction after stroke because of the ability to dynamically monitor the reaction changes occurring at spatial and temporal levels (Frechou et al., 2013). The immune response after stroke is a significant factor that affects the pathobiology and prognosis of acute ischemic stroke (Anrather and Iadecola, 2016). Microglia/macrophages are the main immune cells in the defense against brain damage (Xiong et al., 2016). These cells release mediators, which may have positive effects on brain repair and neurogenesis. Furthermore, superabundant proinflammatory mediators can cause secondary neuronal injury and hinder brain regeneration (Kim et al., 2016).

CD200 and its receptor CD200R are transmembrane glycoproteins present on the surface of cells. Studies have shown that CD200 is expressed on the surface of a wide variety of cells such as thymocytes, B cells, T cells, tonsil follicles, kidney glomeruli, syncytiotrophoblasts, and endothelial cells (Wright et al., 2001). Indirect immunoperoxidase staining showed that CD200 is primarily distributed in all neurons of the spinal cord and brain in the central nervous system (Webb and Barclay, 1984). CD200R is expressed to a lesser extent than CD200 and is mainly distributed in myeloid cells (Wright et al., 2003). In the central nervous system, CD200R is mainly expressed in microglia (Hoek et al., 2000). CD200R1, a member of the CD200R family, binds to CD200 with a higher affinity than other CD200R family members (Gorczynski et al., 2004). Recently, we have focused our attention on the relationship between CD200 and CD200R to elucidate their immunoregulatory functions in neuroinflammation.

Neurons are thought to have a self-protection mechanism in which they express CD200 that binds to CD200R on the microglial surface, which further prevents secondary neuronal injury caused by microglia (Hoek et al., 2000; Wang, 2010; Manich et al., 2019). Although there are no studies on the specific repair mechanisms and therapeutic effects of CD200-CD200R in poststroke inflammation injury, CD200R, could still be considered as a high potential target in the study of stroke immunotherapy.

## Neuroinflammation After Stroke

The innate immune system plays a role in the cerebral damage due to ischemia, hemorrhage, and other brain injuries, and inflammatory signal transduction is involved in all periods of stroke, from the early stages where it mainly causes damage to the late regenerative processes responsible for brain tissue repair (Iadecola and Anrather, 2011). After stroke, primary brain damage results from the death of cerebral cells. Secondary brain damage is caused by cytoplasmic substances released into the extracellular environment, which initiate a cascade of inflammatory events that can amplify cell damage (Wang, 2010). Microglia have profound effects on neuroinflammation. Activated microglia act as a double-edged sword. These cells are normally responsible for clearing up necrotic neural cells and restoring neuronal functions. However, when overly activated after stroke, they produce a high amount of proinflammatory mediators that can destroy the blood-brain barrier and neurons and affect neurogenesis (Xiong et al., 2016). In this review, we have mainly focused our attention on the proinflammatory functions of microglial activation closely linked to the regulator CD200-CD200R.

### Mechanisms of Microglial Activation

During the occurrence of acute brain injuries, microglia shift their activated states depending on two factors: the expression of “on signals” and/or the abnormity of “off signals” (Manich et al., 2019). The “On” signal is principally found in pathological states and involves purines, chemokines, matrix metalloproteinase-3, and glutamate. The “Off” signal mainly appears in the healthy brain and is regulated by the release of CD22, CX3CL1, neurotrophins, and neurotransmitters from neural cells, which could combine with receptors on microglia and help microglia to function in the physiological process (Biber et al., 2007).

Immediately after the occurrence of cerebral injuries, microglia become activated through signals. Damage-associated molecular patterns (DAMPs), which include modified extracellular matrix components, modified or oxidized lipid species, DNA, RNA, and cytoplasmic proteins, are released from the cell intracellular structures at the appropriate time of cell death (Matzinger, 2002). DAMPs activate families of scavenger receptors and Toll-like receptors on microglia, thereby triggering stroke-induced neuroinflammation. In particular, Toll-like receptor 4 (TLR4) and TLR2 are considered to mediate the inflammatory response involved in the pathophysiological processes of cerebral ischemia-induced injury (Kong and Le, 2011). Mice lacking TLR4 showed less expression of mediators relevant to brain damage and inflammation, including IFN-β, COX2, inducible nitric oxide synthase (iNOS), interferon regulatory factor-1(IRF1), and matrix metalloproteinase-9 (MMP-9) (Caso et al., 2007). Moreover, ATP from injured neurons, a type of DAMPs, is a contributing factor that causes microglia to function by binding to the purinergic receptors on microglia (Rodrigues et al., 2015).

### Proinflammatory Functions of Microglial Activation

Once microglia are activated, they undergo major changes in their morphology, functions, and behaviors, such as migration, proliferation, and phagocytosis. The production of proinflammatory cytokines such as IL-6, IL-1, and TNF-α is one of the most crucial abilities of microglia (Zhou et al., 2014). Furthermore, activated microglia synthesize iNOS, which is essential to produce NO. High levels of NO damage the brain under the oxidative stress state because of the oxidation and nitro-tyrosination of useful substances (Guix et al., 2005). On the one hand, the proinflammatory cytokines lead to neuronal death through direct or indirect pathways, for instance, apoptosis and necrosis regulated by the caspase family of proteins and inflammasome (Lamkanfi and Dixit, 2014; Jimenez Fernandez and Lamkanfi, 2015). On the other hand, the proinflammatory cytokines could mediate the phagocytic activity of microglia. After ischemic stroke, higher levels of TNF-α expression were detected in accordance with higher phagocytic activity of microglia (Ritzel et al., 2015). TNF-α has strong immune activity and neurotoxicity (Shichita et al., 2012).

## Role of the CD200-CD200R Interaction in Neuroinflammation

One of the most useful endogenous immunoregulatory molecule candidates that could prevent the neuroinflammation status of the brain tissue seriously altered in cerebral injuries is the CD200/CD200R inhibitory immune ligand-receptor crosstalk. The immunomodulatory effect of CD200-CD200R is produced in the different processes of microglial functions, as shown in the following text.

### Impact on Microglial Proliferation

In this segment, the proliferation of microglia probably affected by the CD200-CD200R interaction is discussed. Deckert et al. (2006) found that in a CD200-deficient mice model with toxoplasma encephalitis, the number of microglia with the proliferation-associated antigen Ki67 + dramatically increased; this finding demonstrates that the deficiency of the CD200-CD200R interaction upregulates microglial proliferation. Furthermore, a study by Wang et al. (2011) showed a high amount of microglia following the exposure to rotenone in the neuron-microglia co-cultures after using an anti-CD200R blocking antibody (ACDR). Oria et al. (2018) observed an increase in the quantity of microglial cells with active phenotype by using a retinoic acid (RA)-induced spina bifida animal model to downregulate CD200 and upregulate CD200R. Thus, it can be speculated that the loss of the CD200-CD200R pair will stimulate microglial proliferation.

### Influence of Microglial Activation

**FIGURE 1:**
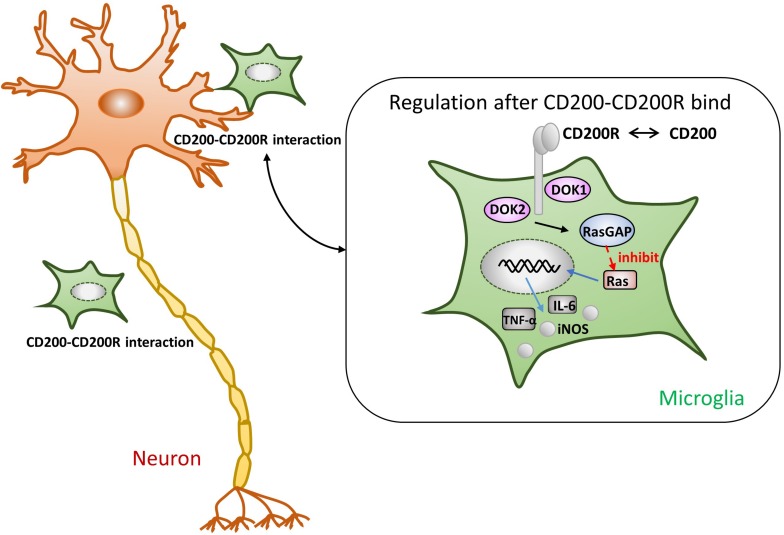
Presumptive mechanism of anti-inflammatory effects through the CD200-CD200R crosstalk. As soon as CD200R binds to CD200, its tyrosine residues are phosphorylated. The tyrosine residues of CD200R recruit the downstream of tyrosine kinase (DOK)1, DOK2, and RasGAP, eventually leading to the inhibition of Ras activation (Manich et al., 2019). Subsequently, the production of TNF-α, IL-6, and iNOS is also suppressed (Hernangomez et al., 2014).

One of the most pivotal roles played by the CD200-CD200R interaction is in maintaining microglia in a resting state to inhibit the release of the proinflammatory mediators. As shown in Figure 1, once CD200R binds to CD200, its tyrosine residues are phosphorylated. Simultaneously, the downstream of tyrosine kinase (DOK)1, DOK2, and RAS p21 protein activator 1 (RasGAP) are recruited. Finally, MAPK p38, extracellular kinase (ERK), and c-Jun terminal kinase (JNK), the common signaling pathways in microglial activation, are suppressed (Manich et al., 2019). Ultimately, the production of TNF-α, IL-6, and iNOS is reduced (Hernangomez et al., 2014). In one study, the authors used mice lacking CD200 to reveal that microglia exhibited a more activated phenotype and were large in numbers (Hoek et al., 2000). Lago et al. (2018) observed that the level of proinflammatory cytokines increased using a selective blocking antibody against CD200R1; this finding indicates that the destruction of CD200R1 induced microglial cells to exhibit a proinflammatory phenotype. Liu et al. (2010) administered a CD200R1 agonist (CD200Fc) in mice with experimental autoimmune encephalomyelitis (EAE) during the chronic phase of the disease; they found that the activation markers of microglia were decreased. To the best of our knowledge, we can predict that the immunomodulatory outcome of CD200-CD200R binding is to inhibit the activation of microglia.

## Role of CD200-CD200R in Stroke

Stroke leads to the death of neural cells. In this condition, the CD200-CD200R has ceased to function. Although understanding the interaction between microglial cells and neurons is a significant challenge, it has a high therapeutic potential to restore damage caused by microglia in neuroinflammation after stroke. For the past few years, various studies have been conducted on CD200-CD200R interaction in stroke in animal models, and satisfactory results have been achieved.

### Effects on Microglial Activation by Mechanisms

Ren et al. (2016) showed CD200 could open the KATP channel (adenosine triphosphate-sensitive potassium) and inhibit the release of ATP as well as the proinflammatory factors in an *in vitro* Parkinson’s disease (PD) model. Thus, it can be said that the CD200/CD200R inhibitory immune ligand-receptor system partially mediates the inhibitory effects of microglial activation by reducing ATP release. Additionally, a report demonstrated that the fusion protein CD200-Fc reduced the levels of TLR4 on the surface of peripherally circulating macrophages in an *in vivo* model of white matter ischemia induced by endothelin-1 (Hayakawa et al., 2016). On the basis of these results, we can speculate that the same process occurs in the microglia. In a recent study, researchers developed animal models with acute stroke by subjecting the wild-type (WT) control mice and CD200R1-knockout (KO) littermate mice to 60 min transient middle cerebral artery occlusion (tMCAO) and assessed post-acute changes in monocyte infiltration, microglia proliferation, and behavioral deficits up to 1 week. Surprisingly, at 72 h after stroke, more deaths occurred in the CD200R1-deficient mice group because of monocyte infiltration and exacerbated microgliosis. On the seventh day, CNS inflammation was resolved in WT mice, whereas microglia activation persisted in CD200R1-KO mice (Ritzel et al., 2019).

### Correlations With Stem Cell Treatment in Stroke

Cell therapy represents a potential breakthrough in the treatment of stroke. Preclinical studies have revealed that cell therapy is effective in the improvement of sensorimotor functions and facilitation of behavioral recovery in animal models of stroke (Mu et al., 2019). More importantly, the putative mechanisms include neuroprotection against inflammation.

In a previous study, the authors used an *in vitro* human allogeneic co-culture model to reveal the interplay between neural stem/progenitor cells (NPCs) and a microglia population. They showed that the proportion of NPCs expressing CD200 and microglia expressing CD200R in the co-culture were higher than those in the mono-culture, leading to the enhanced possibility of the CD200 ligand-receptor binding to ameliorate the detrimental neuroinflammation mediated by microglia (Liu et al., 2013). In addition, Kong et al. (2018) proved that human placenta amniotic membrane-derived mesenchymal stem cells (AMSCs), transplanted into the rat model with ischemic stroke, drastically reduced the level of proinflammatory cytokines accompanied by upregulation of the CD200 protein and suppressed microglia activation compared to the control group. Furthermore, several experiments confirmed that transplantation of various stem cells had anti-inflammatory effects in the treatment of stroke, although the results did not explicitly state that the regulation of inflammation was induced by CD200-CD200R because of the inadequate analysis of CD200 distributed on the surface of stem cells (Borlongan et al., 2010; Eckert et al., 2015; Yoo et al., 2016). It is, however, still reasonable to believe that there is a relationship between the two on a theoretical basis. More research studies are needed to gain further knowledge on this topic.

## CD200-CD200R in Other Neuronal Injuries

The EAE model is used to investigate the relationship between CD200-CD200R and multiple sclerosis (MS). In the experiment, the phenomenon of reduction in CD200 and an increase in CD200R1 was observed in the EAE model. More specifically, CD200 expression showed an apparent reduction before the appearance of clinical symptoms in EAE. This perhaps indicates that changes in CD200 expression might occur in the early phase of MS, which may be the reason for the downregulated control of macrophage/microglial activation, thus contributing to the inflammatory response and the development of pathological processes. In comparison, a succedent increase in CD200R1 expression followed behind that seemingly portended compensatory reaction to re-build control of the inflammation (Valente et al., 2017).

The CD200-CD200R pair also acts as a contributing factor to neurodegenerative diseases such as PD and Alzheimer’s disease (AD). After preinjection of a CD200R blocking antibody to block the CD200-CD200R inhibition signal in rats, a sub-lethal dose of 6-hydroxydopamine, which only caused slight death of dopaminergic neurons in the substantia nigra, resulted in apparent PD symptoms in rats. Histopathological examination showed the death of dopaminergic neurons and activation of microglia cells in the substantia nigra (Zhang et al., 2011). Quantitative studies demonstrated significantly less mRNA and protein levels of CD200 and CD200R in the inferior temporal gyrus and hippocampus (brain regions that show significant AD pathology) from the neuropathologically confirmed AD samples; however, a similar result was not observed in cerebellum samples – an encephalic region that is spared of AD pathology in general (Walker et al., 2009).

## Conclusion and Future Prospects

Recently, CD200 and CD200R have been increasingly investigated, and the immune regulation induced by the CD200-CD200R axis is valuable. As we already know, the toxic damage caused to neurons by overactivated microglia far outweighs its benefits (Weinstein et al., 2010; Kanazawa et al., 2017). Nonetheless, neurons have a self-protection mechanism in which they express CD200 that binds to CD200R on the microglia surface, leading to the negative regulation of proinflammatory factors derived from microglia (Ritzel et al., 2019). Although there are no studies on the specific repair mechanisms and therapeutic effects of CD200-CD200R in poststroke inflammation injury, CD200R can still be considered a high potential target in the study of stroke immunotherapy.

In accordance with the evidence reviewed earlier, stem cells are optimal materials for the cell therapy of patients with stroke. Many animal trials have been conducted, in which the anti-inflammatory effects of stem cells are hot topics (Liu et al., 2013; Eckert et al., 2015; Kong et al., 2018). Yet, some questions remain unsolved. Which kind of stem cell can convert into neurons and express more CD200? Will a combination of stem cells be more effective or less effective? Do stem cells have other signaling pathways that could help enhance the anti-inflammatory regulation of CD200-CD200R interaction? Can we find a method to make the surviving neurons compensate for the high expression of CD200? Or is it possible to use the fusion protein CD200-Fc as a method to treat the harmful neuroinflammation induced by microglia?

In conclusion, compared to traditional treatment regimens, the use of the CD200-CD200R pair as target sites to alleviate poststroke inflammatory injury is certainly one of the best options (Hernangomez et al., 2014). Regulation of microglia using the CD200-CD200R crosstalk meets our natural body rhythm in an even better way and enables our body to conduct efficient signal transmission and maintain the cells in a steady state. Considering this, it is a long and uphill journey to reveal more unknown details about the CD200-CD200R axis.

## Author Contributions

HS conceived the review. JL participated in drafting the manuscript. All authors wrote the manuscript.

## Conflict of Interest Statement

The authors declare that the research was conducted in the absence of any commercial or financial relationships that could be construed as a potential conflict of interest.
